# Vehicular Air Pollution in Houston, Texas: An Intra-Categorical Analysis of Environmental Injustice

**DOI:** 10.3390/ijerph16162968

**Published:** 2019-08-18

**Authors:** Michel G. Loustaunau, Jayajit Chakraborty

**Affiliations:** Department of Sociology & Anthropology, University of Texas at El Paso, El Paso, TX 79968, USA

**Keywords:** environmental justice, air pollution, cancer risk, transportation, Houston

## Abstract

This article contributes to distributive environmental justice (EJ) research on air pollution by analyzing racial/ethnic and related intra-categorical disparities in health risk from exposure to on-road hazardous air pollutants (HAPs) in Harris County, Texas. Previous studies in this urban area have not examined intra-ethnic heterogeneity in EJ outcomes or disproportionate exposure to vehicular pollutants. Our goal was to determine how the EJ implications of cancer risk from exposure to on-road HAP sources differ across and within each major racial/ethnic group (Hispanics, non-Hispanic Blacks, and non-Hispanic Whites), based on data from the Environmental Protection Agency’s National-Scale Air Toxics Assessment (2011) and American Community Survey (2009–2013). Statistical analyses are based on generalized estimating equations which account for clustering of analytic units. Results indicated that Hispanics and non-Hispanic Blacks are exposed to significantly higher cancer risk than non-Hispanic Whites. When each racial/ethnic group was disaggregated based on contextually relevant characteristics, individuals who are in poverty, foreign-born, renters, and have limited English proficiency are found to be disproportionately located in areas exposed to significantly higher cancer risk, regardless of their major racial/ethnic designation. Our findings underscore the need to conduct intra-categorical EJ analysis for uncovering inequalities that get concealed when broadly defined racial/ethnic categories are used.

## 1. Introduction

Under the rubric of distributive environmental justice (EJ) research, numerous quantitative studies have shown that socially disadvantaged groups such as racial/ethnic minorities and individuals of lower socioeconomic status are disproportionately exposed to outdoor air pollution and related health risks [1,2,3,4,5,6,7,8]. Understanding the EJ impacts of hazardous air pollutants has become increasingly important because if socially disadvantaged residents who already experience a compromised health status due to material deprivation and psychosocial stress also face the highest exposure, this exposure exerts larger effects on their health than it does on the average population [9,10,11]. Additionally, socially disadvantaged individuals often reside in older or damaged homes that contain more entry points for outdoor air pollutants (e.g., cracks, gaps, and openings in walls and windows), making them more susceptible to poor health [12]. Conversely, socially advantaged individuals are less likely to suffer adverse health effects because they can afford to equip their dwellings with air purification [11], purchase newer homes, and/or reside in neighborhoods with lower exposure to air pollution.

While EJ scholarship on air pollution has documented various social injustices and continues to expand in new directions, we focus here on one particular limitation that has received limited attention in previous research. Most distributive EJ studies conducted in the U.S. have used broad racial/ethnic categories to define minority populations groups (e.g., percent Black or percent Hispanic) and analyze racial/ethnic inequities in exposure to environmental risks. Such broad racial/ethnic categorizations ignore within-group differences and assume a level of homogeneity within minority populations that may not exist in most U.S. urban areas or nationally. An intra-categorical approach to EJ analysis that addresses this issue was first introduced by Collins et al. [13] in their study of unequal air pollution health risks in El Paso, Texas, based on locally relevant socio-demographic variables. Their results demonstrated how Hispanic ethnic status interacts with class, gender, and age to amplify health risks within the Hispanic group. Subsequent EJ scholarship has emphasized the need to acknowledge diversity and heterogeneity within the Hispanic category, especially in immigrant gateway cities such as Miami, Florida [14,15]. These studies of Hispanic heterogeneity found significantly higher levels of exposure to vehicular air pollutants for specific Hispanic subgroups in the Miami metropolitan area (e.g., Cubans, foreign-born, and unemployed Hispanics). While intra-categorical EJ research has focused mainly on Hispanics, a few recent studies have extended this approach to examine other racial/ethnic groups. A national level analysis of the Asian population reported intra-ethnic differences in cancer risk from air pollutants [16]. Specifically, risk burdens were significantly greater in neighborhoods with higher percentages of Chinese, Korean, and South Asians, as well as non-English speaking Asians, compared to the corresponding White percentage. Another EJ study found significantly greater cancer risks from both mobile and stationary air pollution sources for Black Americans residing in economically deprived neighborhoods [17]. More comparative and systematic analyses are required to document intra-categorical disparities in EJ outcomes and understand the different ways in which racial/ethnic status combines with other social characteristics in contributing to unequal exposure to air pollution risks. Factors such as income, education, nativity, language proficiency, homeownership, and age can interact differently with race/ethnicity to amplify or attenuate risk inequalities for specific subgroups within the broader racial/ethnic categories. Our paper addresses this growing need to provide new insights on the role of intra-ethnic heterogeneity in shaping patterns of environmental injustice, especially in U.S. urban areas where this issue has not been examined before.

This study seeks to contribute to distributive EJ research through a detailed examination of intra-ethnic differences in potential health risks from exposure to on-road sources of hazardous air pollutants (HAPs) in Harris County, Texas. Harris is the most populous county in Texas, located in the Greater Houston Metropolitan Statistical Area (MSA). The Houston area has been the focus of several early and groundbreaking EJ studies that focused on the unfair siting of hazardous waste sites in Black communities [18,19]. More recent EJ studies have reported significantly higher levels of cancer risk from exposure to air pollutants for Hispanic and non-Hispanic Black populations in Harris County and other counties of the Greater Houston MSA [20,21,22,23]. However, previous EJ research has not examined intra-ethnic heterogeneity in exposure to environmental risks in this urban area, or attempted to examine the EJ consequences of air pollution emitted by vehicular (mobile) sources.

Our study examines how the EJ implications of cancer risks from ambient exposure to HAPs emitted by on-road sources differ across and within each major racial/ethnic group (i.e., Hispanics, non-Hispanic Blacks, and non-Hispanic Whites) by disaggregating each group on the basis of contextually relevant social characteristics. The primary research question to be investigated is as follows: is estimated cancer risk from inhalation exposure to HAPs from on-road sources distributed inequitably with respect to race and ethnicity, after controlling for socioeconomic and contextual factors? Our specific focus is to examine how the answer to this research question, or the EJ implications of cancer risk from on-road HAP emissions, differ when: (1) each major racial/ethnic category (i.e., Hispanic, non-Hispanic Black, and non-Hispanic White) is treated as a single group, following most previous EJ studies; and (2) each major racial/ethnic category is subdivided into contextually relevant subgroups based on six specific characteristics: poverty status, nativity, homeownership, educational attainment, language proficiency, and age. This study combines modeled estimates of potential cancer risk from the U.S. Environmental Protection Agency (USEPA)’s 2011 National-Scale Air Toxics Assessment with socio-demographic data from 2009–2013 American Community Survey (ACS) five-year estimates. Statistical analyses are based on multivariate generalized estimating equation (GEE) models, which account for clustering of census tracts in Harris County and provide more statistically valid inferences regarding the social determinants of exposure to on-road cancer risks compared to traditional regression models commonly used in prior EJ research.

## 2. Materials and Methods 

This section describes the data sources, variables, and analytic approaches used to assess the EJ implications of cancer risk from exposure to on-road HAP sources in Harris County, with a focus on intra-ethnic differences in EJ outcomes.

### 2.1. Study Area

**Figure 1:**
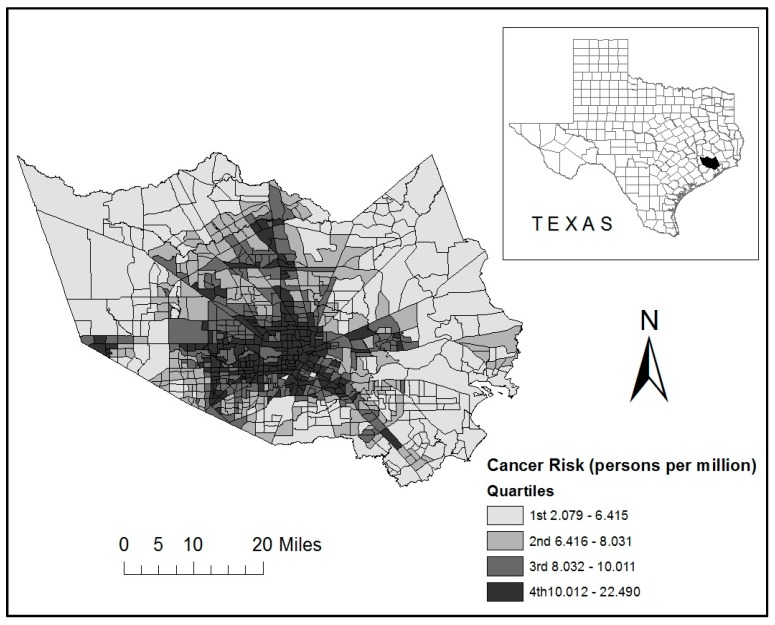
Distribution of on-road lifetime cancer risk by census tract in Harris County, Texas.

Harris County is located in the Greater Houston MSA in southeastern Texas, as shown in Figure 1. With a population of approximately 4.5 million residents (2013), Harris is the largest county in Texas and the third largest in the U.S. The county seat is Houston—the largest city in Texas and fourth largest nationally. According to the 2013 ACS estimates, Hispanics (41%), non-Hispanic Whites (33%), and non-Hispanic Blacks (19%) represent the three largest racial/ethnic groups in this county.

With regards to EJ research, Harris County is a particularly appropriate study area because of its racial/diversity and air pollution problems that adversely affect the health and well-being of residents. A report authored by the *Mayor’s Task Force on Health Effects of Air Pollution* concluded that air pollution levels in Houston are considered to be unacceptable by knowledgeable experts and local residents, and an important cause of several respiratory and cardiopulmonary health effects [24]. Vehicular air pollution has emerged as a serious health concern for people residing in Harris County. Tailpipe emissions from cars, trucks, and buses were identified by the Mayor’s Task Force as one of the most important source categories for air pollution health risks in this urban area. On-road emissions from motorized vehicles have been linked to significant increases in daily traffic volumes in the last decade [25]. Potential cancer risks from cumulative HAP exposure have been found to be significantly greater in neighborhoods containing higher proportions of minorities and lower socioeconomic status individuals in this urban area [20,21,22,23]. Previous EJ studies have only used broadly defined racial/ethnic categories (e.g., percent Hispanic and percent non-Hispanic Black) and not focused specifically on vehicular air pollution—two limitations that our research seeks to address.

### 2.2. Dependent Variable: Cancer Risks from On-Road Sources of Hazardous Air Pollutants

The Clean Air Act of 1990 separated air pollutants into two distinct categories: criteria air pollutants and hazardous air pollutants (HAPs). HAPs, also known as air toxics, include 187 specific substances that are known to cause cancer and other serious health effects, such as developmental and respiratory problems, damage to the immune system, as well as neurological and reproductive problems [26]. To measure health risks from exposure to vehicular sources of air pollutants, we used the U.S. EPA’s National-Scale Air Toxics Assessment (NATA), which has emerged as an important database for estimating health risks associated with the inhalation of HAPs, as well as the most reliable data source for spatially explicit characterization of HAP risk exposure in U.S. urban areas [22,25,27,28,29].

Our study utilizes the 2011 NATA, the fifth national assessment that includes estimates of ambient exposure concentrations for 180 of the 187 Clean Air Act HAPs and diesel particulate matter. The 2011 NATA encompasses a four-step process to develop the assessment for estimating cancer risks at the census tract level. The first step is to compile the national emissions inventory from outdoor sources, the second step is to estimate ambient concentrations of air toxics, the third step is to generate exposure concentrations, and the fourth step is to identify possible health risks associated with the inhalation of air toxics. The result of the USEPA’s four-step process is a database containing modeled estimates of cancer and non-cancer risks. This information can be downloaded at the national, state, county, and census tract levels [30].

The 2011 NATA estimates potential cumulative risks to public health from HAP exposure following the USEPA’s risk characterization guidelines that assume a lifelong exposure to 2011 levels of emissions. Cancer risks in the 2011 NATA are based on unit risk estimates, which represent an individual’s probability of contracting cancer from a lifetime of exposure to a concentration of one microgram of the pollutant per cubic meter of air. For each census tract, the individual lifetime cancer risk (LCR) associated with each HAP is calculated by combining exposure concentration estimates with available unit risk estimates and inhalation reference concentrations. Cancer risks associated with various HAPs are assumed to be additive and are summed to compute an aggregate LCR for each tract, measured in persons per million. An LCR of 1 in a million, for example, implies that 1 out of one million equally exposed people would contract cancer if exposed continuously (24 h per day) to that specific concentration over a lifetime (70 years). This would be an excess LCR, in addition to other cancer cases that would normally occur in an unexposed population of one million people [30].

The 2011 NATA cancer risk estimates for this study were obtained directly from the USEPA NATA website for all census tracts (based on 2010 U.S. Census boundaries) in Harris County, Texas. The dependent variable for this study is represented by estimates of LCR (persons per million) associated with inhalation exposure to HAPs released only from on-road mobile sources. On-road sources in the NATA include those that operate on roads and highways for transportation of passengers or freight, and include cars, trucks, buses, and motorcycles [30].

### 2.3. Independent Variables

Socio-demographic variables for this study were extracted from the census tract level 2009–2013 American Community Survey (ACS) five-year estimates, since this spatial resolution and timeframe provided the best match with the 2011 NATA cancer risk data. To ensure reliable proportion estimates and adequately high denominators for all independent variables, tracts with a population of less than 500 were not included. Previous EJ studies in Harris County and Greater Houston had also excluded tracts with less than 500 people [21,23,31]. This led to the exclusion of two tracts; the remaining 784 tracts were used for our analysis. Our study population thus included 4,123,688 persons, out of which 41.7% were Hispanic, 18.8% were non-Hispanic Black, and 33.0% non-Hispanic White.

To examine the effect of race and ethnicity, we included separate variables that collectively cover the entire population: the proportion of individuals self-identifying to be of Hispanic/Latino origin (of any race), non-Hispanic Black, non-Hispanic White, non-Hispanic Asian, and non-Hispanics belonging to a race other than White, Black, or Asian (i.e., American Indian or Alaskan Native, Pacific Islander, or some other race). Following prior EJ studies, population density (persons per square mile) was included as a control variable based on the assumption that densely populated areas are more likely to contain air pollution-generating activities or roadways which increase cancer risks for residents [3,16,20,21,32]. Median household income was also used as an additional control variable, since areas with higher air pollution health risks are associated with lower income levels in most previous EJ studies [13,16,33]. Summary statistics for the dependent variable, major racial/ethnic groups, and control variables are provided in Table 1.

The six characteristics that are used to disaggregate the three major racial ethnic/categories include poverty status, nativity, homeownership, educational attainment, language proficiency, and age. The proportion of individuals below poverty level (i.e., family income below federal poverty line) was selected since previous studies using the NATA have found that exposure to HAPs in the U.S. are more likely to occur in areas with a higher proportion of people in poverty [3,13,20]. Nativity (i.e., U.S. born vs. foreign born) was considered since this variable is known to be associated with social disadvantage for Hispanics in the U.S. which could be also the case for non-Hispanic Whites and non-Hispanic Blacks [14,34]. Homeownership (i.e., owner-occupied vs. renter-occupied housing units) was chosen since this variable is linked with wealth, political engagement, and participation in local decision making [21,22]. Educational attainment (i.e., grade level less than high school vs. high school diploma or higher) was selected because limited access to educational opportunities and advancement is associated with social disadvantage, as well as higher exposure to air pollution risks [13]. Language proficiency (i.e., the ability to speak English very well) was selected, as the inability to communicate in English could influence a person’s ability to participate in decisions that affect chronic environmental exposure. Language barriers are also associated with isolation from the broader community and linguistic isolation was found to be positively correlated with air pollution risks in Greater Houston [21]. Finally, age status was also chosen to distinguish between younger (i.e., those who are less than 65 years old) and older (those who are aged 65 years or more) residents. The only intra-categorical EJ study that subdivided Hispanics and non-Hispanic Whites on the basis of age [13] used the same classification. Previous EJ studies have also used age over 65 years as one of the variables to formulate indicators of economic insecurity and neighborhood instability [23,35], or define transportation-disadvantaged individuals [3].

These six characteristics are assumed to be significant contributors to the heterogeneity that exists among the three largest racial/ethnic groups in Harris County, and potentially lead to differences in how environmental injustices are experienced within each group. While the Hispanic category has been disaggregated using some of these characteristics in previous intra-categorical EJ research, non-Hispanic White and non-Hispanic Black categories have not been unpacked systematically in this fashion, except for [13]. Summary statistics for the variables used to subdivide the Hispanic, non-Hispanic Black, and non-Hispanic White categories are provided in Table 2.

### 2.4. Statistical Methodology

To analyze the EJ implications of cancer risk from on-road sources of HAPs in Harris County, we specified generalized estimating equations (GEEs) with robust covariance estimates. GEEs extend the generalized linear model to the analysis of clustered data, while relaxing several assumptions of traditional regression models, including normality of variable distribution [36,37]. In order to fit a GEE, clusters of observations must be specified, assuming that observations within a cluster are correlated and observations from different clusters are independent. We defined clusters using 2009–2013 ACS estimates for median year of housing construction, based on the following categories: “2000 or later”, “1990 to 1999”, “1980 to 1989”, “1970 to 1979”, “1960 to 1969”, “1950 to 1959”, “1940 to 1949”, and “1939 or earlier”. This yielded eight clusters of census tracts for our study area. This cluster definition approach was chosen because it corresponds with spatial and temporal dimensions of the built environment that are linked to the formation of environmental injustices [22,38]. This cluster definition has also been used in recent EJ studies conducted in the Houston area that utilized GEEs [22,25,31,39]. A GEE also requires the specification of an intra-cluster dependency correlation matrix, referred to as the working correlation matrix [36]. For this purpose, all GEEs were modeled with different correlation structure specifications available in IBM SPSS Statistics software, using the quasi-likelihood under the independence criterion (QIC) goodness-of-fit coefficient to determine the best working correlation specification [22]. The QIC tests indicated that the ‘exchangeable’ specification performed better than all other specifications of the working correlation matrix for only two GEE models, while the ‘unstructured’ specification performed best for all remaining GEEs. These specifications were thus used for the GEEs presented here.

To select the most appropriate GEE, several model specifications were explored. Since the dependent variable (cancer risk) was continuous, we examined the normal, gamma, and inverse Gaussian distributions. For each of these distributions, GEEs based on both identity and log link functions were explored. An identity link function assumes the dependent variable is predicted directly and not transformed, while a log link function implies that the natural logarithm of the dependent variable is predicted. The inverse Gaussian distribution with the log link function was finally selected for all GEEs, since this specification yielded the lowest value of the QIC, indicating the best statistical fit. All independent variables were standardized and standardized coefficients are provided in the tables summarizing the GEE results. To check for multicollinearity, the multicollinearity condition index (MCI) was calculated for the combination of all independent variables included in the GEE models. The MCIs for all models were found to range from 3.0 to 7.0, indicating the absence of serious collinearity problems among the standardized explanatory variables.

The first phase of the study follows a conventional approach based on most previous EJ studies, where each major racial/ethnic category (i.e., Hispanic, non-Hispanic Black, and non-Hispanic White) was treated as a single group. In the second phase, three different sets of models were used to separately disaggregate each of these major racial/ethnic groups based on six characteristics: poverty (proportion above and below federal poverty level), nativity (proportion U.S. born and foreign-born), homeownership (proportion owners and renters), educational attainment (proportion high school or higher and proportion less than high school), language (proportion proficient in English and limited English proficiency), and age (proportion age below 65 years and age of 65 years or more). For each racial/ethnic category, six GEE models were thus needed to examine and compare each disaggregated subgroup to its counterpart subgroup (reference variable). In each of these models, the socially advantaged subgroup (i.e., above poverty, U.S. born, homeowners, high school or higher, English proficient, and age below 65) was treated as the reference category, to allow direct comparison with the subgroup expected to be socially disadvantaged (i.e., below poverty, foreign born, renters, less than high school diploma, limited English proficiency, aged 65 or more years). All multivariate models include population density and median household income as control variables, as well as the two other racial/ethnic categories that were not disaggregated.

## 3. Results

The spatial distribution of LCR from on-road HAP sources at the tract level is displayed in Figure 1. In this map, the lightest shading is used to display tracts in the lowest quartile (bottom 25%) of on-road cancer risk and darkest shading is used for tracts in highest quartile (top 25%). Tracts with highest values are located mainly in central Harris County, including the city of Houston. In contrast, tracts with the lowest risk are located in the peripheral areas of the county. Cancer risk values tend to decrease as the distance from downtown Houston increases.

### 3.1. Traditional EJ Analysis

The results of multivariate GEE analysis using broad racial/ethnic categories are summarized in Table 3. In all three models, on-road cancer risk is significantly and positively related to population density and median household income (*p* < 0.05), after controlling for other variables and clustering. Model 1 indicates significantly lower proportions of non-Hispanic Blacks and non-Hispanic Whites, compared to the Hispanic proportion (reference variable) in tracts with greater on-road cancer risk. Model 2 indicates a significantly higher proportion of Hispanics and lower proportion of non-Hispanic Whites, compared to the non-Hispanic Black proportion (reference variable) in tracts with greater on-road cancer risk. Finally, model 3 indicates a significantly higher proportion of both Hispanics and non-Hispanic Blacks, compared to the non-Hispanic White proportion (reference variable) in tracts with greater on-road cancer risk. The proportion of non-Hispanic Asians is significant and positive in all three GEE models, which suggests relatively higher cancer risk burdens for this group.

In summary, our multivariate EJ analysis based on traditional or broadly defined racial/ethnic categories provides strong evidence of distributive injustices for on-road HAP exposure. With respect to the non-Hispanic White proportion, the proportions of Hispanics and non-Hispanic Blacks are significantly greater in tracts exposed to higher cancer risks from vehicular HAP sources. The Hispanic proportion shows the strongest positive association with cancer risk, when compared to the proportions of non-Hispanic Blacks or Whites. The next phase of the study disaggregates the three largest racial/ethnic categories to examine whether specific subgroups within each category are facing disproportionately higher cancer risks in Harris County.

### 3.2. Intra-Categorical EJ Analysis

The results of the multivariate GEE analysis for specific subgroups associated with each of three major racial/ethnic categories are summarized in Table 4, Table 5 and Table 6, respectively. In each GEE model or table column, the subgroup expected to be more socially disadvantaged is directly compared to its counterpart subgroup, which is treated as the reference variable. The results for each major racial/ethnic group are discussed separately in the following paragraphs.

Table 4 presents results for GEEs with on-road cancer risk as the dependent variable and disaggregates the Hispanic category in six different ways (models 1 to 6). Model 1 indicates significantly higher on-road cancer risk for Hispanics below poverty compared to those above poverty (reference variable), after controlling for the effects of other independent variables and clustering. Similar results can be observed in models 2 to 6, where the proportion of the tract population who are Hispanic and foreign born, renters, less educated, less proficient in English, and above 65 years of age, show significantly higher cancer risks compared to their counterpart or reference subgroups. The coefficients for the overall non-Hispanic Black and non-Hispanic White proportions are significantly smaller when compared to those of socially advantaged Hispanic subgroups (except for Hispanics above poverty).

Table 5 presents results from GEEs with on-road cancer risk as the dependent variable and disaggregates the non-Hispanic Black category (models 1 to 6). Model 1 indicates significantly higher on-road cancer risk for non-Hispanic Blacks below the poverty line compared to those above poverty (reference variable), after controlling for the effects of other independent variables. Similar results can be observed in models 3, 4, and 6, where the proportion of non-Hispanic Blacks who are renters, less educated, and are 65 years of age, show significantly higher cancer risks compared to their counterpart subgroups. Models 2 and 5, however, indicate significantly lower on-road cancer risk for the proportions of non-Hispanic Blacks who are foreign born compared to those who are U.S. born (reference variable), and those who are less proficient in English compared to those who are English proficient (reference variable), after controlling for the other variables. The coefficients for the overall Hispanic proportion are significantly higher when compared to those of socially advantaged non-Hispanic Black subgroups above the poverty line, are U.S. born, and own homes. In all six models, the coefficients for the overall non-Hispanic White proportion are negative and significant, which suggests higher cancer risk for socially advantaged non-Hispanics Blacks compared to the non-Hispanic White population.

Table 6 presents GEEs with on-road cancer risk as the dependent variable and disaggregates the non-Hispanic White category (models 1 to 6). With the exception of model 4, all GEEs indicate significantly higher on-road cancer risk for socially disadvantaged non-Hispanic Whites (i.e., who are below the poverty line, foreign born, renters, less proficient in English, and aged above 65 years), when compared to their counterpart subgroups. In all six models, the coefficients for the overall Hispanic and non-Hispanic Black proportions are significant and positive, which suggests higher cancer risk for these racial/ethnic minority groups compared to the socially advantaged White residents of Harris County.

## 4. Discussion

Our study examined two research questions associated with disproportionate exposure to HAPs from on-road emission sources in Harris County, Texas. The first question focused on investigating if estimated cancer risks from HAP exposure are distributed inequitably when each major racial/ethnic category is treated as a single homogenous group, after controlling for relevant factors and geographic clustering. Multivariate GEE analysis revealed that the proportion of both Hispanics and non-Hispanic Blacks are significantly higher than the non-Hispanic White proportion, in tracts exposed to greater on-road cancer risk from HAP exposure. The Hispanic proportion was also found to be even higher than the non-Hispanic Black proportion in tracts facing greater cancer risk. Overall, the results suggest that racial/ethnic minorities are disproportionately distributed with respect to cancer risk from vehicular HAPs sources in Harris County, with Hispanics facing greater risk burdens compared to non-Hispanic Black and White residents. These results are similar to those reported by other quantitative EJ studies in the Houston area that have found similar distributive injustices for cumulative cancer risk associated with exposure to all HAPs [20,21,22,23].

The second research question focused on investigating whether cancer risks from exposure to on-road HAPs are distributed inequitably when each major racial/ethnic category is subdivided into contextually relevant subgroups. This specific question was answered by disaggregating each major racial/ethnic group based on six socio-demographic characteristics. Multivariate GEE results indicate that Hispanics subgroups facing significantly greater on-road cancer risk compared to their counterpart subgroups include those who are below the poverty line, foreign born, renters, lack high school education, have limited English proficiency, and are 65 or more years old. Non-Hispanic Blacks exposed to significantly greater on-road cancer risk than their counterparts comprise those who are below the poverty line, renters, less educated, and are 65 or more years old. Non-Hispanic Whites facing significantly greater on-road cancer risk when compared to their counterpart subgroups include those who are below the poverty line, foreign born, renters, less proficient in English, and above 65 years old.

Our multivariate analysis of intra-ethnic heterogeneity thus confirms that both Hispanic and non-Hispanic Black residents are prone to experience a form of ‘multiple jeopardy’ [11,13] in which their disadvantaged racial/ethnic status interacts in significant ways with poverty, education, homeownership, and age status to amplify experiences of unequal exposure to vehicular air pollution risks. For Hispanics, being foreign-born and having limited English proficiency are additional factors that contribute to greater cancer risk burdens. Our results for several Hispanic subgroups are particularly disturbing, since socially disadvantaged Hispanics who are facing disproportionately higher on-road cancer risk are also more likely to lack health insurance and other resources necessary to mitigate the adverse health effects of toxic air pollution. Previous research suggest that Hispanics have the lowest health insurance rate of any racial/ethnic group in the U.S. and this disparity is significantly higher for Hispanics [40,41].

Our findings for non-Hispanic Whites differ from those reported in the only intra-categorical EJ study to disaggregate the White population [13]. Although this study did not use multivariate analysis or examine on-road HAP sources, no significant intra-group disparities in cancer risk were found when non-Hispanic Whites were subdivided by age, education, or poverty status in El Paso County, Texas. Given the significantly positive associations observed for most socially disadvantaged non-Hispanic White subgroups in Harris County, our results do not indicate that White racial status interacts with income, homeownership, or age to reduce cancer risk disparities. However, on-road cancer risks associated with the broader non-Hispanic White category was found to consistently and significantly lower than those faced by socially advantaged Hispanic and non-Hispanic Black subgroups, which partially supports the assertion that “Anglo-whiteness articulates as a protective factor” [13] (p. 344) for on-road HAP exposure.

It is important to recognize some of the limitations of the data and methodology utilized in this study. Although estimates of on-road cancer risk from the 2011 NATA published by the USEPA are used to represent the dependent variable for this study, there are specific limitations with this dataset [30]. The 2011 NATA estimates cancer risk only from direct inhalation exposure to outdoor air pollutants and does not account for exposure through other pathways, such as ingestion or skin contact, as well as exposure to HAPs produced indoors, such as emissions from cars in attached garages. The NATA information is also not a substitute for actual health outcomes or cancer incidence data, and only represents modeled estimates of cancer risk based on EPA’s risk assessment guidelines. Additionally, the NATA risk estimates only include individual and additive health effects, but synergistic interactions among HAPs could pose additional cancer risks that are not examined in this study [30].

Finally, it is important to consider that this study focused on evaluating the current patterns of on-road HAP exposure and related distributional injustices, and not the processes that led to the observed racial/ethnic disparities. Since we did not utilize longitudinal data, our results cannot be used to explore causal mechanisms or infer the sequence of events that caused increased exposure to on-road HAPs in tracts within Harris County that are disproportionately populated by specific socially disadvantaged subgroups. However, Figure 1 indicates that cancer risks from vehicular HAPs are substantially higher in central Houston, compared to the suburban areas of the county. While racial/ethnic minority residents have traditionally resided in the inner urban core, suburban neighborhoods have undergone rapid transformation and integration as central Houston has gentrified in recent years [42]. As high-income White households in this county have abandoned the suburbs for the inner city, our results reflect that socioeconomically advantaged Black and Hispanic subgroups are relocating to suburban tracts with lower exposure to traffic pollution. More research is necessary to identify the historical trajectories of highway and suburban development, changing patterns of racial/ethnic migration, gentrification and urbanization, and other political, socioeconomic, and spatial processes in this metropolitan area that are potentially responsible for the unequal distribution of vehicular air pollution risks. Future research should also consider the disaggregation of the Asian category, since this group is facing significantly higher exposure to on-road cancer risk compared to other racial/ethnic categories. Recent EJ studies focusing on the Asian population [16,43] provide an important framework for more detailed intra-categorical analysis necessary to determine which social characteristics are related to the environmental injustices currently experienced by Asian Americans in Harris County.

## 5. Conclusions

This article has sought to contribute to distributive environmental justice (EJ) scholarship by analyzing racial/ethnic inequities and related intra-categorical disparities in ambient exposure to on-road sources of HAPs in Harris County, Texas. Most quantitative EJ studies have not disaggregated traditional racial/ethnic categories, and the few multivariate studies that have done so have only disaggregated one specific category, such as Hispanics. Our study thus addresses an important gap by conducting a comparative and comprehensive analysis of intra-categorical differences in estimated cancer risks associated with on-road HAP exposure for three major racial/ethnic groups. Since previous EJ scholarship in the Houston area has used broad racial/ethnic designations and has not analyzed risk disparities associated with vehicular air pollutants, this study also contributes new empirical knowledge regarding environmental injustices in this urban area.

Our statistical results provide strong evidence of environmental injustices in Harris County, since cancer risk from on-road HAP exposure is disproportionately distributed with respect to the racial and ethnic characteristics of the population. When treated as a single group, non-Hispanic Black and Hispanic residents are significantly more likely to reside in neighborhoods with higher cancer risk from HAP exposure compared to non-Hispanic Whites, after controlling for population density, income, and spatial clustering. With regards to intra-ethnic heterogeneity, our findings demonstrate that all subgroups within these broader racial/ethnic categories are not equally exposed to on-road cancer risks. Socially disadvantaged subgroups are disproportionately located in neighborhoods exposed to significantly higher cancer risk, regardless of their racial/ethnic designation. Within all three major categories (i.e., Hispanics, non-Hispanic Blacks, and non-Hispanic Whites), the subgroups facing significantly higher risk include individuals in poverty, foreign born persons, renters, and those with limited English proficiency. For these disadvantaged subgroups, even their White racial status does not reduce the risk burdens associated with on-road HAP exposure. In order to better understand distributive environmental injustices and contribute to the formulation of policies that address unequal exposure to air pollution risks in Houston and other urban areas, it is necessary to incorporate intra-ethnic heterogeneity more explicitly in future EJ research and policy.

## Figures and Tables

**Table 1:** Descriptive statistics for key variables (N = 784 tracts).

Variables	Min	Max	Mean	Std. Dev.
On-road cancer risk (persons per million)	2.078	22.490	8.368	2.862
Proportion Hispanic	0.001	0.973	0.407	0.256
Proportion non-Hispanic Black	0.000	0.953	0.191	0.218
Proportion non-Hispanic White	0.000	0.940	0.326	0.267
Proportion non-Hispanic Asian	0.000	0.461	0.060	0.071
Proportion non-Hispanic other race	0.000	0.055	0.001	0.005
Population density	52	67,718	5123	4159
Median household income ($)	12,272	250,000	58,586	34,736

**Table 2:** Descriptive statistics for variables used in intra-categorical analysis (N = 784 tracts).

Variables	Min	Max	Mean	Std. Dev.
Hispanic:				
Proportion above poverty	0.044	1.000	0.571	0.249
Proportion below poverty	0.000	0.955	0.428	0.249
Proportion U.S. born	0.000	1.000	0.604	0.138
Proportion foreign born	0.000	1.000	0.395	0.138
Proportion homeowners	0.000	1.000	0.522	0.295
Proportion renters	0.000	1.000	0.477	0.295
Proportion high school or higher	0.000	1.000	0.623	0.217
Proportion less than high school	0.000	1.000	0.376	0.217
Proportion English proficient	0.166	1.000	0.636	0.178
Proportion limited English proficient	0.000	0.833	0.363	0.178
Proportion age below 65 years	0.676	1.000	0.954	0.045
Proportion age of 65 or more years	0.000	0.478	0.050	0.057
Non-Hispanic Black:				
Proportion above poverty	0.000	1.000	0.762	0.242
Proportion below poverty	0.000	1.000	0.237	0.242
Proportion U.S. born	0.000	1.000	0.921	0.127
Proportion foreign born	0.000	1.000	0.078	0.127
Proportion homeowners	0.000	1.000	0.410	0.337
Proportion renters	0.000	1.000	0.589	0.337
Proportion high school or higher	0.000	1.000	0.877	0.163
Proportion less than high school	0.000	1.000	0.122	0.163
Proportion English proficient	0.000	1.000	0.974	0.082
Proportion limited English proficient	0.000	1.000	0.025	0.082
Proportion age below 65 years	0.000	1.000	0.916	0.137
Proportion age of 65 or more years	0.000	1.000	0.083	0.137
Non-Hispanic White:				
Proportion above poverty	0.000	1.000	0.809	0.157
Proportion below poverty	0.000	1.000	0.190	0.157
Proportion U.S. born	0.000	1.000	0.929	0.094
Proportion foreign born	0.000	1.000	0.070	0.094
Proportion homeowners	0.000	1.000	0.649	0.265
Proportion renters	0.000	1.000	0.350	0.265
Proportion high school or higher	0.000	1.000	0.896	0.135
Proportion less than high school	0.000	1.000	0.103	0.135
Proportion English proficient	0.160	1.000	0.763	0.192
Proportion limited English proficient	0.000	0.839	0.236	0.192
Proportion age below 65 years	0.000	1.000	0.813	0.141
Proportion age of 65 or more years	0.000	1.000	0.186	0.141

**Table 3:** Multivariate generalized estimating equation (GEE) analysis of on-road lifetime cancer risk (LCR): Beta coefficients. QIC = quasi-likelihood under the independence criterion.

Variables	Model 1	Model 2	Model 3
Population density	0.254 **	0.253 **	0.250 **
Median household income	0.046 *	0.046 *	0.048 *
Proportion Hispanic	Ref	0.035 **	0.153 **
Proportion non-Hispanic Black	−0.027 *	Ref	0.103 **
Proportion non-Hispanic White	−0.158 **	−0.122 **	Ref
Proportion non-Hispanic Asian	0.103 **	0.113 **	0.148 **
Proportion non-Hispanic other race	−0.017	−0.016	−0.014 *
Intercept	2.129 **	2.128 **	2.126 **
Scale	0.017	0.017	0.017
Model fit (QIC)	24.953	24.965	24.699

* *p* < 0.05; ** *p* < 0.01; N = 784.

**Table 4:** Intra-categorical GEE analysis for the Hispanic population: Beta coefficients.

Variables	Model 1	Model 2	Model 3	Model 4	Model 5	Model 6
Population density	0.268 **	0.235 **	0.210 **	0.265 **	0.248 **	0.258 **
Median household income	0.061 **	0.029	0.087 **	0.051 *	0.046 *	0.030 *
Proportion Non-Hispanic Black	−0.004	−0.027 *	−0.045 **	−0.022 *	−0.018 *	−0.021 *
Proportion Non-Hispanic White	−0.116 **	−0.125 **	−0.164 **	−0.116 **	−0.112 **	−0.148 **
Proportion Non-Hispanic Asian	0.108 **	0.106 **	0.062 **	0.119 **	0.128 **	0.096 **
Proportion Non-Hispanic other	−0.013 *	−0.021 *	−0.013 *	−0.015 *	−0.015 *	−0.016 *
Proportion Hispanic: above poverty	Ref					
Proportion Hispanic: below poverty	0.065 **					
Proportion Hispanic: U.S. born		Ref				
Proportion Hispanic: foreign born		0.058 **				
Proportion Hispanic: homeowners			Ref			
Proportion Hispanic: renters			0.134 **			
Proportion Hispanic: high school or higher				Ref		
Proportion Hispanic: less than high school				0.061 **		
Proportion Hispanic: English proficient					Ref	
Proportion Hispanic: not English proficient					0.068 **	
Proportion Hispanic: age below 65 years						Ref
Proportion Hispanic: age of 65 or more years						0.042 **
Intercept	2.140 **	2.123 **	2.143 **	2.136 **	2.136 **	2.124 **
Scale	0.016	0.016	0.013	0.016	0.016	0.016
Model fit (QIC)	26.181	24.976	27.257	24.395	26.042	22.354

* *p* < 0.05; ** *p* < 0.01; N = 784.

**Table 5:** Intra-categorical GEE analysis for the non-Hispanic Black population: Beta coefficients.

Variables	Model 1	Model 2	Model 3	Model 4	Model 5	Model 6
Population density	0.215 **	0.256 **	0.186 **	0.260 **	0.252 **	0.249 **
Median household income	0.047 **	0.037 *	0.082 **	0.037 *	0.041 *	0.038 *
Proportion Hispanic	0.023 *	0.032 *	0.040 *	0.025	0.027	0.024
Proportion non-Hispanic White	−0.430 **	−0.454 **	−0.482 **	−0.431 **	−0.470 **	−0.451 **
Proportion non-Hispanic Asian	1.950 **	1.687 **	2.184 **	1.650 **	1.599 **	1.562 **
Proportion non-Hispanic other	−3.970 *	−2.208	−2.690 *	−4.216 *	−2.766 *	−2.873 *
Proportion non-Hispanic Black: above poverty	Ref					
Proportion non-Hispanic Black: below poverty	0.266 **					
Proportion non-Hispanic Black: U.S. born		Ref				
Proportion non-Hispanic Black: foreign born		−0.192 *				
Proportion non-Hispanic Black: homeowners			Ref			
Proportion non-Hispanic Black: renters			0.232 **			
Proportion non-Hispanic Black: high school or higher				Ref		
Proportion non-Hispanic Black: less than high school				0.202 **		
Proportion non-Hispanic Black: English proficient					Ref	
Proportion non-Hispanic Black: not English proficient					−0.154 *	
Proportion non-Hispanic Black: age below 65 years						Ref
Proportion non-Hispanic Black: age of 65 or more years						0.132 *
Intercept	2.068 **	2.187 **	1.997 **	2.144 **	2.187 **	2.166 **
Scale	0.017	0.017	0.017	0.017	0.017	0.017
Model fit (QIC)	24.486	27.348	31.967	25.683	26.161	25.202

* *p* < 0.05; ** *p* < 0.01; N = 784.

**Table 6:** Intra-categorical GEE analysis for the Non-Hispanic White population: Beta coefficients.

Variables	Model 1	Model 2	Model 3	Model 4	Model 5	Model 6
Population density	0.244 **	0.221 **	0.254 **	0.238 **	0.213 **	0.237 **
Median household income	0.058 **	0.047 **	0.072 **	0.048 **	0.042 **	0.048 **
Proportion Hispanic	0.137 **	0.132 **	0.119 **	0.149 **	0.060 *	0.138 **
Proportion non-Hispanic Black	0.085 **	0.099 **	0.072 **	0.116 **	0.052 **	0.112 **
Proportion non-Hispanic Asian	0.150 **	0.086 **	0.056 **	0.137 **	0.166 **	0.143 **
Proportion non-Hispanic other	−0.014 *	−0.017 *	−0.023 **	−0.016 *	−0.012	−0.016 *
Proportion non-Hispanic White: above poverty	Ref					
Proportion non-Hispanic White: below poverty	0.042 **					
Proportion non-Hispanic White: U.S. born		Ref				
Proportion non-Hispanic White: foreign born		0.119 **				
Proportion non-Hispanic White: homeowners			Ref			
Proportion non-Hispanic White: renters			0.097 **			
Proportion non-Hispanic White: high school or higher				Ref		
Proportion non-Hispanic White: less than high school				−0.015		
Proportion non-Hispanic White: English proficient					Ref	
Proportion non-Hispanic White: not English proficient					0.120 **	
Proportion non-Hispanic White: age below 65 years						Ref
Proportion non-Hispanic White: age of 65 or more years						0.016 *
Intercept	2.120 **	2.137 **	2.141 **	2.125 **	2.120 **	2.122 **
Scale	0.017	0.015	0.013	0.016	0.017	0.017
Model fit (QIC)	24.274	23.168	21.535	24.470	25.348	23.834

* *p* < 0.05; ** *p* < 0.01; N = 784.
